# Effects of Five Coumarins and Standardized Extracts from *Tagetes lucida* Cav. on Motor Impairment and Neuroinflammation Induced with Cuprizone

**DOI:** 10.3390/ph16101391

**Published:** 2023-09-30

**Authors:** Gabriela Castro-Martínez, Maribel Herrera-Ruiz, Manases González-Cortázar, Sandra Liliana Porras-Dávila, Julio Cesar Almanza Pérez, Enrique Jimenez-Ferrer

**Affiliations:** 1 Doctorate in Biological and Health Sciences, Metropolitan Autonomous University, Mexico City 14387, Mexico; gcm_19@hotmail.com; 2 Southern Biomedical Research Center, Mexican Social Security Institute, Guadalajara 44340, Mexico; cibis_herj@yahoo.com.mx (M.H.-R.); gmanases2000@gmail.com (M.G.-C.); davilasp1117@gmail.com (S.L.P.-D.); 3 Department of Health Sciences, Division of Biological and Health Sciences, Universidad Autónoma Metropolitana-Iztapalapa, Mexico City 09340, Mexico

**Keywords:** multiple sclerosis, cytokines, 7-Isoprenyloxycumarin, (IC), herniarin, (HN), 7-O-Prenylscopoletin, (PE), dimethylfraxetin, (DF), scoporone (SC)

## Abstract

Multiple sclerosis (MS) is an autoimmune disease of the central nervous system (CNS) with no curative treatment, and the available therapies aim to modify the course of the disease. It has been demonstrated that extracts of *Tagetes lucida* have immunomodulatory and neuroprotective effects. This work induced motor damage and neuroinflammation in male BALB/c mice by oral administration of cuprizone (CPZ) (40 mg/kg) for five weeks. In addition, the extracts and coumarins of *Tagetes lucida* (25 mg/kg) were used to control these damage variables; during the experiment, animals were subject to behavioral tests, and at the end of 5 weeks, mice from each group were used to measure the integrity of biological barriers (brain, kidneys, and spleen) through the extravasation test with blue Evans dye. In another group of animals, the ELISA method measured the brain concentrations of IL-1β, IL-4, IL-10, and TNF-α. The results presented here allow us to conclude that the extracts and coumarins IC, HN, PE, DF, and SC of *Tagetes lucida* exert a neuroprotective effect by controlling the motor damage and neuroinflammation by increasing the expression of IL-4 and IL-10 and decreasing IL-1β and TNF-α; notably, these treatments also protect organs from vascular permeability increase, mainly the BBB, in mice with CPZ-induced experimental encephalomyelitis (VEH * *p* < 0.05). However, more studies must be carried out to elucidate the molecular mechanisms of the pharmacological effects of this Mexican medicinal plant.

## 1. Introduction

Multiple sclerosis (MS) is a chronic autoimmune disease of the central nervous system (CNS), characterized by inflammation and neurodegeneration associated with the demyelination of neurons [1,2]. Myelin is produced by oligodendrocytes, resident cells of the CNS, where a single oligodendrocyte can myelinate up to 80 axon segments to facilitate axonal signaling [3].

MS is mediated by altering the immune response in genetically predisposed individuals [2,4]. This alteration is characterized by multiple focal lesions that affect the CNS myelin sheaths and generate a series of clinical symptoms caused mainly by the neuroinflammatory and demyelinating process, which leads to axon damage [5]. The etiology of MS is unknown; however, epidemiological data indicate that genetic and environmental factors such as virus infections (Epstein–Barr), smoking, vitamin D deficiency, latitude, and a high body mass index could influence the development and progression of the disease [2,4,6]. MS is a disabling neurological disease acquired in children, adolescents, and adults and is diagnosed by magnetic resonance imaging (MRI). MS generates psychological, economic, and social problems, affecting approximately 2.5 million people worldwide [4,7,8,9]. Its clinical manifestations begin in the third and fourth decades of life. It develops preferably in women, with a female/male ratio of 3:1 [8,10]. In terms of the clinical course, there are several MS subtypes: relapsing-remitting (RR), with relapses of disease separated by periods without clinical progression; secondary progressive (SP), which represents the phase of the disease when a gradual neurological deterioration (progression) follows a period of RR disease; primary progressive (PP), affecting approximately 15% of people with MS in whom the neurological deterioration is present from the onset; and the progressive-remitting (PR) subtype in which a few acute exacerbations are superimposed on the gradual PP-like course and which is considered the less frequent subtype of MS [7]. MS develops from infiltrating autoreactive T cells and other immune system cells through the blood–brain barrier (BBB) into the CNS, causing significant damage [1,10,11,12]. Four critical pathological features of MS have been described. (a) Inflammation is generally believed to be the main trigger of events leading to CNS damage, although it has recently been suggested that initial damage to neuroglial elements may trigger secondary inflammation in some cases. (b) Demyelination, the hallmark of MS, is where the inflammatory process destroys the myelin sheath or oligodendrocyte cell body. (c) Axonal loss or damage is the third feature and the fourth (d) is gliosis, an astrocytic reaction due to damage to the CNS [7,11]. MS is a highly complex disease due to the variety of clinical pictures that can occur depending on the area of the CNS that is affected, whether it is the brain or the spinal cord, and which are specific to each patient, depending on the individual’s particular set of motor, sensory or cognitive disorders [2]. Although MS is a disease that only affects humans, the search for new pharmacological treatments has led to the development of animal models, both in vitro and in vivo, which attempt to reproduce some of the clinical manifestations of the disease. Within the in vivo models, we find the cuprizone (CPZ)-induced intoxication model. It has been shown that this compound is capable of causing temporary demyelination and reproducing some manifestations in mice similar to those that occur in the clinic in human patients [13,14]. CPZ (Bis(ciclohexanona)oxaldihidrazona) is a copper chelator that induces apoptosis of oligodendrocytes (cells that produce myelin) during its administration, which leads to demyelination of different brain areas such as the corpus callosum, hippocampus, basal ganglia, and cerebellum [3,14,15]. This demyelination initiates motor disability and an exacerbated accumulation of microglia and astrogliosis that promotes an inflammatory response within the first three weeks of CPZ feeding [3,14,16]. The CPZ model widely reported in the literature consists of the administration of 0.2% CPZ mixed with powdered food for rodents for 5 to 6 weeks, thus generating acute demyelination, or for 12 weeks for chronic demyelination [14]. There is currently no curative treatment for MS [11]. Instead, the available therapies aim to modify the disease’s course and control its symptoms, controlling episodes of neuroinflammation [1,6].

Therefore, it is necessary to research new therapeutic alternatives that are safe and effective. Several plants that have been essential instruments of traditional medicine in different pathologies are now being used as new alternatives. *Tagetes lucida* “pericón” taxonomically belongs to the Asteraceae family; it is a plant used in traditional medicine to treat gastrointestinal problems involving colic, ulcers, diarrhea, dysentery, indigestion, vomiting, typhoid fever, and urogenital problems. Additional conditions treated include rheumatic pain, asthma, varicose veins, inflammation, abortion, carminative activity, anthelmintic infection, musculoskeletal pain, and neurological and inflammatory diseases [17,18,19]. In addition, *Tagetes lucida* (*T. lucida*) has been used for the treatment of emotional and nervous disorders as part of a mixture with other anxiolytic plants, disorders characterized by a “state of physical and mental restlessness” capable of diminishing the ability to achieve daily goals [18,20,21,22]. Recent studies have shown great interest in the basic research and application of *T. lucida*, and several authors have demonstrated the biological activity of this species in different diseases. Pharmacological studies on the use of *T. lucida* have indicated antioxidant, antibacterial, and anti-inflammatory properties, and in addition to influencing the activity of the CNS, this species is useful as an antinociceptive, antidepressant, anxiolytic antipsychotic, and sedative [14,15,16,18,20,21,22,23,24,25]. 

This work aimed to evaluate the effect of standardized extracts and coumarins obtained from *T. lucida* in a model of chemically induced MS involving the chronic administration of cuprizone in BALB/c mice and quantifying neurological behavior with the Irwin test, motor impairment, neuroinflammation (IL-1β, TNF-α, IL-4, and IL-10), and vascular permeability in different organs (brain, kidneys, spleen). 

## 2. Results

### 2.1. Quantification and Chemical Identification of Five Coumarins

**Figure 1:**
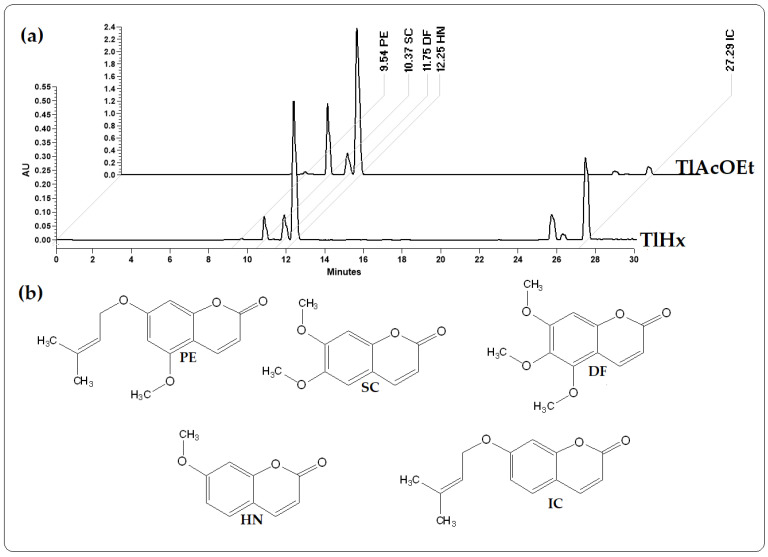
Chromatograms from HPLC analysis: (**a**) extracts from *T. lucida*, TlHx and TlAcOEt, at 330 nm; (**b**) chemical structures of 7-O-Prenylscopoletin (PE), Scoporone (SC), Dimethylfraxetin (DF), Herniarin (HN), and 7-Isoprenyloxycumarin (IC).

High-performance liquid chromatography (HPLC) was used to investigate the extracts obtained using hexane (TlHx) and ethyl acetate (TlAcOEt) and identify different compounds. Figure 1 is a chromatogram that compares the two extracts at a wavelength of 330 nm. The results revealed the presence of five coumarins that correspond, according to their retention times (RT) and UV spectra, to 7-Isoprenyloxycoumarin (IC) with an RT of 27.29 min (λ = 204.0, 322.0 nm), herniarin (HN) with an RT of 12.25 min (λ = 219.2, 322.0 nm), dimethylfraxetine (DF) with a 11.75 min RT (λ = 215.7, 294.7, 335.3 nm), scoporone (SC) with an RT of 10.37 min (λ = 229.7, 294.8, 343.4 nm) and 7-O-prenylscopoletin (PE) with an RT of 9.54 min (λ = 219.7, 324.4 nm). These compounds were analyzed by 1H, and 13 NMR and the data were compared against standards and those described in the literature (Supplementary Data) [22].

The identified compounds (in µg/mg of extract) were quantified using data from the standard curve of each compound in HPLC. Table 1 shows the concentrations of each coumarin. As shown in Figure 1, the size of the peaks of each compound is different in each extract, which was verified with the quantification, with 7-Isoprenyloxycumarin (IC) being more concentrated in the less polar extract (TlHx). At the same time, Herniarin (HN)), Dimethylfraxetin (DF), Scoporone (SC), and 7-O-prenylscopoletin (PE) are found in higher concentrations in TlAcOEt. It should be noted that in both extracts, the SC is the most concentrated coumarin.

### 2.2. Behavioral Tests

#### 2.2.1. The Irwin Test

The animals were observed for five weeks, noting the Irwin parameters. Table 2 shows the percentage of mice that presented piloerection in each group. No changes occurred in any groups in the first week of CPZ administration.

In the second week with CPZ, 20% of the VEH group already had piloerection; subsequently, the number of animals that presented this symptom increased over time so that by the fourth and fifth weeks, there were already 93% of individuals presenting symptoms. In the group that received silymarin (SLM), it was also observed that the percentage of individuals with piloerection was higher over time. However, the drug prevented the value from being higher than that of the VEH group. By the fifth week, only 60% of mice presented piloerection. Prednisone (PDN), an anti-inflammatory steroid, also prevented piloerection elevation in the mice, compared with VEH, and by the fifth week, the percentage was 26%. TlHx or TlAcOEt caused a lower percentage of animals to bristle compared with the VEH group from the third week of treatment, and by the end of the experiment (fifth week), it was observed in only 20% of mice in each group. All coumarin compounds resulted in significant protection against piloerection throughout the five weeks. The group that received PE did not present piloerection in the second week and up to the third week, with a percentage of 40% that decreased until the fifth week when only 6% of individuals showed the symptom.

Moreover, SC also protected the mice, and already in the last week, the same percentage of only 6% presented piloerection. Another parameter observed during the Irwin test was piling (Table 3), which was present from the first week in all the groups analyzed, except in healthy animals. The percentage of individuals who presented crowding behavior due to the administration of CPZ in the VEH group gradually increased until reaching 100% in the fifth week. The administration of SLM or PDN caused the animals to avoid crowding for five weeks during the experiment. A high percentage of animals showed crowding behavior, but in the end, only 20% of individuals presented this behavior. The animals in the groups that received one of the *T. lucida* treatments all avoided crowding, a large decrease compared with the behavior caused by the CPZ without treatment. It should be noted that, mainly in the fifth week, TlAcOEt, PE, and DF did not cause any of the mice in these groups to present this behavior.

#### 2.2.2. Open Field Test (OFT)

**Figure 2:**
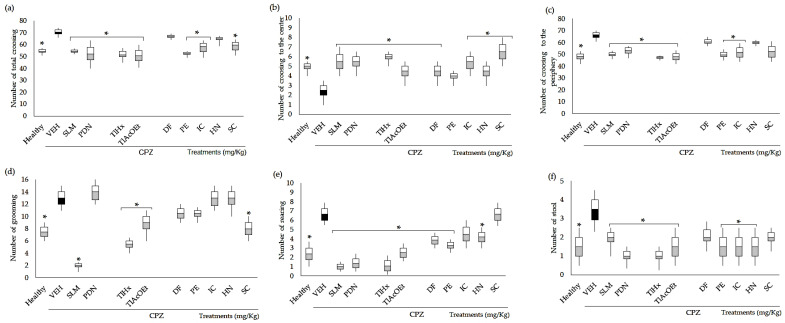
Effect of *T. lucida* in the open field test on BALB/c mice administered with CPZ at the end of week five. (**a**) Number of total crossings. (**b**) Number of crossings to the center. (**c**) Number of crossings to the periphery. (**d**) Number of grooming behaviors. (**e**) Number of rearing behaviors. (**f**) Number of stools. Data were analyzed with ANOVA followed by Dunnett’s test (mean ± SD n = 8), and the difference was considered significant when * *p* < 0.05 in comparison with the VEH group.

The CPZ caused a significant increase in the total crossings in the open field (Figure 2a), in the number of crossings in the periphery (Figure 2c), in grooming (Figure 2d), in rearing (Figure 2e), and in the frequency of stool (Figure 2f); additionally, this compound provoked a significant decrease in the number of crosses in the center (Figure 2b) when compared with the group of healthy animals (* *p* < 0.05). All the treatments administered, including the control drugs (SLM and PDN) and the *T. lucida* products, significantly decreased the number of total crossings and increased the crossings to the center compared with VEH (* *p* < 0.05), except HN and DF, which did not affect the total crossovers (*p* > 0.05) and PE, which did not show significant changes in the number crossovers in the center in comparison with VEH (Figure 2a,b). 

Considering the number of crossings to the periphery, the standard drugs, the extracts TlHx and TlAcOEt, and the coumarins IC and PE significantly reduced the number of events caused by the CPZ (* *p* < 0.05, Figure 2c). At the same time, mice treated with HN, DF, and SC did not change this behavior in the OFT. Parameters associated with a behavioral disturbance in mice exposed to a stressful environment, such as grooming, rearing, and stool, were modified in different ways compared with VEH. Thus, the number of grooming behaviors was significantly decreased with the administration of SLM, TlHx, TlAcOEt, and SC (* *p* < 0.05, Figure 2d). SLM, PDN, TlHx, TlAcOEt, DF, HN, and PE, decreased the rearing and stool when data were compared with the VEH group (* *p* < 0.05, Figure 2e,f). However, DF only actively reduced the rearing, and SC did not modify these two variables (*p* > 0.05). 

#### 2.2.3. Rota-Rod Test (Rr)

**Figure 3:**
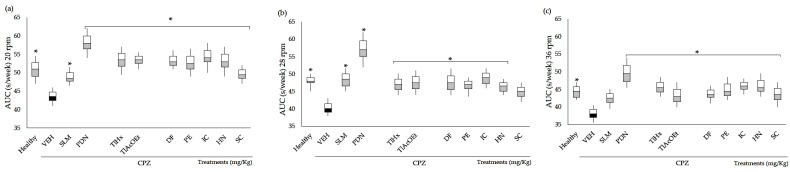
AUC of motor coordination of mice BALB/c administered with CPZ for five weeks and exposed to the rota-rod (Rr) test. Three different speeds: (**a**) 20 rpm, (**b**) 28 rpm, and (**c**) 36 rpm. Data are presented as mean ± SD, with n = 8, * *p *< 0.05 indicates significant differences between groups in comparison with the VEH group, by using ANOVA with the post-test of Tukey.

In the present study, the Rr test indicated a deficit in motor activity (gross motor skills) in the VEH group, as in the Franco-Pons test. Figure 3 shows the area under the curve (AUC) of residence time for the data obtained during the five experimental weeks. The animals were evaluated in their motor coordination behavior in the Rr test at 4, 12, 20, 28, and 36 rpm; the data of 4 and 12 rpm do not show significant changes in comparison with VEH (VEH *p* > 0.05). The deterioration in motor coordination caused by CPZ was observed starting at 20 rpm and continued up to 28 and 36 rpm (Figure 3a–c), with a significant difference between the healthy and VEH groups (* *p* < 0.05). However, as can be seen in Figure 3, all the groups of animals treated with SLM, PDN, TlHx, TlAcOEt, and coumarins showed reduced damage, all of them being statistically different from VEH (* *p* < 0.05), except SC at 28 rpm and SLM at 36 rpm, which only showed a tendency for increased AUC without being different from the VEH values. 

### 2.3. Effect of Extracts and Coumarins from T. lucida on Vascular Permeability

**Figure 4:**
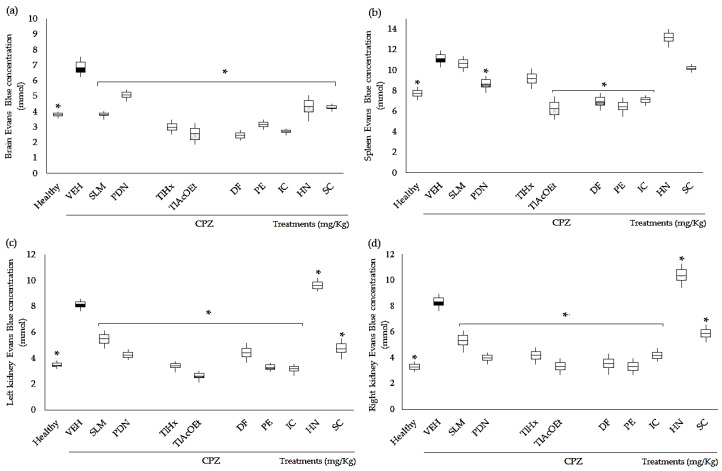
Effect of extracts and coumarins from *T. lucida* on vascular permeability in the brain, the kidneys, and the spleen from mice with CPZ treatment. Optical density was measured at 630 nm, and the measurements were converted into µmol of dye extravasated using the EB extinction coefficient ε = 7.81 × 10^4^. (**a**) Brain, (**b**) spleen, (**c**) left kidney, and (**d**) right kidney. Data are presented as mean ± SD, with n = 4, * *p *< 0.05 indicates significant differences between groups compared with the VEH group using the Student *t*-test.

CPZ caused a significant increase in the concentration of Evans blue (EB) extravasated in the brain, spleen, and kidneys, in comparison with the group of healthy animals (* *p* < 0.05), which indicates an increase in the permeability of the capillary system of each organ, and the BBB in the case of the brain. This effect was significantly controlled downward in almost all organs by all *T. lucida* treatments and control drugs, and the data are different from the VEH results (Figure 4, * *p* < 0.05); however, there were some exceptions; for example, neither SLM, TlHx, HN, nor SC could control extravasation in the spleen. At the same time, in the kidneys, HN was significantly different from that of the VEH group, but because it increases the permeability of these organs even more (Figure 4c,d; * *p* < 0.05).

### 2.4. Effect of Extracts and Coumarins from T. lucida on Cytokine Concentrations

**Figure 5:**
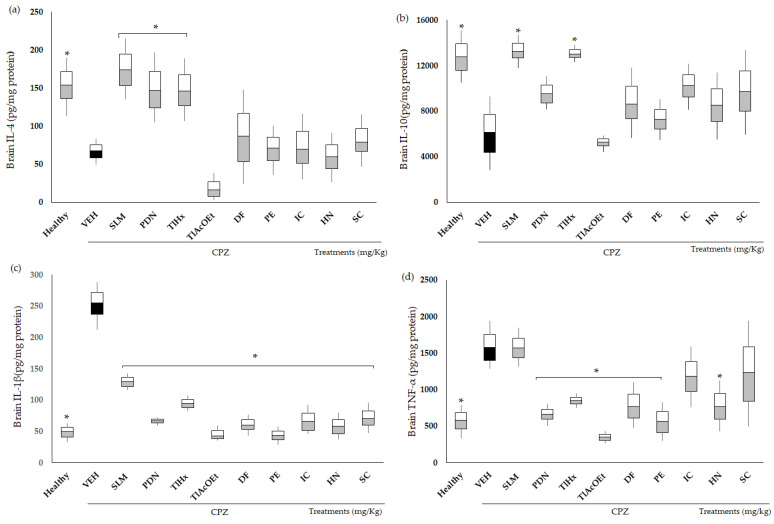
Effect of extracts and coumarins from *T. lucida* on the concentrations of pro- and anti-inflammatory cytokines in the brain. (**a**) IL-4, (**b**) IL-10, (**c**) IL-1β, and (**d**) TNF-α. Data are presented as mean ± SD, with n = 6, * *p* < 0.05 indicates significant differences between groups compared with the VEH group, using ANOVA followed by Tukey Test. SLM = silymarin, PDN = prednisone.

The inflammation associated with the chronic administration of CPZ was quantified by measuring the concentrations of anti- and pro-inflammatory cytokines (Figure 5). CPZ caused a decrease in the concentration of IL-4 and IL-10 in the brains of mice, whereas increases in TNF-α and IL-1β were observed. All data were statistically different from the group of healthy animals (* *p *< 0.05). The drugs used as the positive control, SLM and PDN, increased the value of IL-4 in the brain, while for IL-10, only SLM induced a significant change compared with VEH (* *p *< 0.05), and PDN only showed a trend. Although not significantly different from the damage group (*p *> 0.05), both treatments decreased IL-1β brain concentrations, and for TNF-α, only PDN modified the effect of CPZ (* *p *< 0.05). 

TlHx was the only treatment from *T. lucida* that induced a significant change compared with VEH (* *p *< 0.05) and increased the concentrations of IL-4 and L-10 in the brain. All treatments from *T. lucida* inhibited increases in IL-1β in the brains of the animals, with values that were significantly different from VEH (* *p *< 0.05). For TNF-α, a similar pattern was observed, with the levels of this pro-inflammatory cytokine decreasing (* *p *< 0.05), except for the animals that received IC and SC.

## 3. Discussion

### 3.1. Quantification and Chemical Identification of Five Coumarins

Recent research has shown that different extracts of *T. lucida* have properties such as anti-inflammatory and antioxidant activities and mainly have a potential use against illnesses related to the CNS, providing antinociceptive and sedative effects, and treatment for disorders of depression and anxiety [17,18,19,21,23,25,26,27,28,29]. The HPLC analysis of the TlHx and TlAcOEt extracts allowed the identification of five coumarins with biological activity: IC, HN, DF, SC, and PE. These coumarins have already been reported for other species besides *T. lucida* [30], although IC and PE have only been identified in this last species [25]. This group of secondary metabolites (coumarins) comprises great diversity; the union of different functional groups forms the basic skeleton of coumarin (2H-chromen-2-one) [31]. Pharmacological studies of these coumarins have demonstrated their potential therapeutic use for CNS diseases as they are considered anti-neuroinflammatory compounds [25,26]. A pharmacokinetic study of a mixture of five coumarins (IC, HN, DF, SC, and PE), administered by the oral pathway (10 mg/kg), was conducted to measure the bioavailability of these compounds as neuroprotectives. The concentration was quantified by an HPLC-DAD-UV method in samples of plasma, brain, kidney, and spleen from healthy mice and animals with neuroinflammation secondary to the administration of LPS. The results showed an increase in the plasma concentration of coumarins up to three times in the group with neuroinflammation compared with the healthy control group. The same was observed in the quantification of coumarins in the brain, thus demonstrating the ability of these compounds to cross the BBB, suggesting this as an alternative treatment for diseases associated with neuroinflammation processes [32].

### 3.2. Evaluation of Two Extracts and Five Coumarins against the Motor Damage Induced by CPZ

The model of chronic and forced administration (per os, p.o.) of cuprizone (CPZ) was used to assess the effect of TlHx, TlAcOEt and the five coumarins on motor damage, BBB permeability, and neuroinflammation. 

The data available in the literature on the CPZ model indicate that the animals consume this substance in their daily diet. However, in the present work, a scheme of forced administration (p.o.) of the toxicant was proposed, which ensures that the administered dose is exact. Thus, the application route is the main difference between this design and those proposed by other researchers [33].

### 3.3. Behavioral Tests

Animals with (sick) or without (healthy) CPZ and animals with damage and subjected various treatments were subjected to different behavioral tests: Irwin test, OFT, and Rr. The Irwin Test is used to evaluate, through observation, behavioral changes or physiological manifestations that allow us to study the effects of substances on the CNS, from doses without observable effects to doses that could induce toxicity [34]. During the Irwin Test, it was observed that the CPZ caused physiological changes such as piloerection and crowding behavior, and these increased over time. It has been established that piloerection is a response of mice exposed to stressful environments. It is regulated by activation of the sympathetic nervous system through physiological mechanisms such as thermoregulation, increased heart rate, increased blood pressure, muscle tone, and excessive sweating. It is caused mainly by different stimuli, such as sadness, fear, pain, threat, and anxiety [35,36]. The pathophysiological frame of EM is derived from the administration of CPZ; this toxin has a stimulant effect, which activates the CNS, causing piloerection secondary to the alteration of the sympathetic nervous system and also causing the overcrowding behavior of the animals. Mice’s snuggling behavior may stem from an attempt to regulate temperature through the social attachment they display in this behavior. The groups treated with *T. lucida* had similar behaviors to the healthy animals since piloerection and curling up occurred at a low percentage compared with the healthy group.

In addition to these results, it was observed that CPZ can cause motor damage associated with the demyelination that it induces. It is probable that CPZ causes these changes in mice and that the administration of plant-derived treatments partially reverses them. The alterations that the mice with CPZ manifested in the Irwin Test agree with what was observed in the open field test (OFT), which is widely used to measure the spontaneous motor behavior of rodents associated with arousal or inhibition of the locomotor system. In addition, the assay has been validated to measure anxiety-like effects since the nature of rodents in a new environment is to seek protected spaces. In this case, the periphery (close to the box walls) represents security. At the same time, the center of the field is sensed as dangerous. Thus, the increase in the number of crossings in the periphery translates as a state of anxiety, while crossings in the center indicate a decrease in anxiety [37]. 

It can be deduced from these behaviors that CPZ can provoke changes associated with locomotion or anxiety, which are behavioral determinants when mice are exposed to the open field apparatus (OFT). Fluctuations in the animals’ locomotion indicate alterations and could denote an abnormal function of the CNS. Another premise related to the behavioral changes observed in this trial is that they can be used to measure the general health of animals. Thus, the sick or stressed mice showed decreased motor capacity when exposed to the OFT but also showed increased grooming or rearing behaviors. Thus, it can be observed that the CPZ causes stress. With increased stress, it was observed that in the OFT, the mice preferred to remain close to the walls and travel more in the periphery area (thigmotaxis), a behavior that increases in situations of injury or stress. 

Furthermore, remaining in the periphery is a sign of increased anxiety-like behavior; conversely, rats with low anxiety tend to spend more time in the center of the field area [38]. This behavior was observed in healthy mice during the experimentation in the present work. These behaviors are likely a consequence of the motor or cognitive damage that CPZ generates by the demyelination of different CNS structures. In addition, it has been established that patients with progressive MS have overstimulation of the SNS due to chronic inflammation [35]. A study by Pérez Ortega et al. in 2016 demonstrated that extracts of different polarities from *T. lucida* had a sedative effect by reducing the spontaneous motor behavior of mice and an anxiolytic effect by interacting with the serotonergic and GABAergic neurotransmission system, attributing the possible effect to compounds identified as DF and flavonoids [18].

Grooming is an innate body-cleaning behavior found in healthy rodents but it can also show repetitive and stereotyped patterns due to CNS disorders such as stress and anxiety. Thus, the increase in grooming, hyperlocomotion, bristling hair, and piling indicate that the VEH group presented an overstimulation of the CNS and probable noradrenergic activation in the limbic system (corpus callosum, thalamus, hypothalamus, hippocampus, amygdala, midbrain), a system responsible for modulating emotions and behavior [35,36,39]. Regarding the ability to restore motor impairment and the emotionality parameters evaluated in the OFT, the most consistent results were observed in the mice treated with the extracts TlHx and TlAcOEt since they counteracted the effects of cuprizone on the six variables analyzed. The chemical composition of both treatments includes the significant presence of coumarins, to which the effects observed in both extracts could be attributed, at least in part. Each compound has a different activity level; thus, IC controls five of the six variables analyzed in the OFT, followed by PE > HN = SC > DF. The coumarins IC and HN are the less complex structures of the five analyzed here, both substituted in position 7, the first with an isoprenyl group and the other with only a methoxyl. If we consider both, it was observed that a more complex radical in that position is better at reversing the damage caused by CPZ; it was also observed that when the compound is more substituted in different positions, the activity in the OFT also decreased.

The effects of the extracts and compounds of *T. lucida* on variables in the OFT is related to their activity on the CNS; thus, for example, in 2015, an antidepressant effect of extracts and fractions of this plant on rats subjected to the forced swimming test was reported [21,23]. Recently, the antipsychotic capacity of the aqueous and ethyl acetate extracts was reported. In addition, the HN and DF obtained from this species, are antipsychotics, which was demonstrated in a model of acute psychosis induced by ketamine through quantifying motor behavior in OFT, depression in the forced swimming test, and memory in the passive avoidance test. In the same work, the interaction of these natural treatments with haloperidol was identified since a potentiation of catalepsy in mice induced by that drug was observed. The results of both trials indicate that these *T. lucida* products modify glutamatergic and dopaminergic neurotransmission; both are involved in the pathophysiology of neurological diseases such as schizophrenia [27]. This finding is interesting since ketamine, also used as an antidepressant, can reverse the effects caused by CPZ. For example, repeated treatment with (R)-ketamine (10 mg/kg/ daily, twice a week, for six weeks) significantly improved demyelination and activated microglia in the brain compared with saline-treated mice [40]. Thus, it can be deduced that *T. lucida* could be exerting its protective effect against the actions of cuprizone due to its ability to interact with the glutamatergic system as ketamine does [27], since the products of the plant interact with that drug. More studies are necessary to establish the relationship between this neurotransmission system and coumarins.

CPZ has been shown to induce behavioral deficits that correlate with the demyelination of different structures, suggesting that behavioral testing may serve as a valuable surrogate marker of the demyelinating event [41]. In 2008, Torkilds reported behavioral deficits in CPZ-treated animals from visual observations, indicating motor dysfunction within five weeks of CPZ treatment, which were monitored in the Rr test [16]. 

The results of the Rr test indicate that the VEH group has low resistance (strength), as well as a lack of balance and coordination, which could represent a peripheral nervous system (PNS) dysfunction, as well as demyelination in CNS structures such as the cerebellum or brainstem and the motor cortex. On the one hand, the cerebellum controls motor force, balance, and coordination, and the brainstem allows communication between the brain and the spinal cord to send signals to the PNS; however, demyelination of these structures would cause communication dysfunction of the axons (synapses) [35,36]. While the locomotion of the groups treated with the SLM and PDN reference drugs and the *T. lucida* treatments differed from the VEH group, these behavioral results are consistent with the evaluations already carried out for this plant, indicating its importance as a treatment for CNS diseases. It should also be noted that the naturally occurring drug SLM, consisting of a mixture of flavonoids from *Silybum marianum*, has a significant effect as a neuroprotector in the CPZ model utilized in the present investigation. Furthermore, this drug has antiviral and anticancer activities, as well as antioxidant and anti-inflammatory effects, and contributes to improving the condition of other diseases such as Alzheimer’s [42,43].

### 3.4. Evans Blue Extravasation

The CPZ modifies the behavior of the animals, simulating the demyelination that occurs during MS. One of the pathophysiological events that it triggers is an increase in the permeability of the blood–brain barrier (BBB) in such a way that the control exerted by the CPZ is altered [44]. Cerebral vasculature restricts the transport of molecules from the blood and CNS parenchyma [45]. Deterioration of the BBB is observed due to infiltration and activation of microglia, which can cause neuroinflammation with an increase in proinflammatory cytokines. Therefore, in this work, the level of extravasation was also measured using the Evans blue (EB) quantification technique in vivo after its intravenous injection. The EB dye has a high affinity for albumin, so this test is based on the fact that under basal physiological conditions, albumin does not cross the BBB [46]. However, under conditions of inflammation, blood vessels “extravasate” proteins, resulting in a blue coloration of tissues that have endothelial damage (increased permeability) [47]. 

In the present work, it was shown that the administration of CPZ (VEH group) allows EB extravasation, indicating an increase in permeability, as has been indicated in the literature [44], which may be due in part to the inflammatory process and the oxidative stress generated in response to oligodendrocyte apoptosis [3]. Apoptosis leads to the release of various cytokines and chemokines that activate other cells, such as astrocytes and microglia, generating a pro-inflammatory environment [14].

The structural composition of the BBB is complex, which makes it possible to protect the brain from exposure to immune cells, neurotoxic substances derived from endogenous metabolites, proteins, or xenobiotics from the environment. For this reason, an alteration in the permeability of the BBB is associated with different stimuli, including inflammation, OXE, and traumatic stress. The present work showed that the treatments of *T. lucida*, SLM, and PDN behaved as neuroprotectors. Furthermore, another research group recently published a paper on a chemical fraction obtained from *T. lucida*. The researchers standardized the content of the five coumarins evaluated here, demonstrating that the 10 mg/kg dose of the said treatment protects the BBB in mice with a toxic stimulus of LPS by decreasing the extravasation of EB [32]. In the case of PDN, an anti-inflammatory drug from the group of glucocorticoids that also acts as an immunosuppressant, inhibiting the activity of phospholipase A2 consequently decreases the release of arachidonic acid from the phospholipids of cell membranes. This action prevents the formation of prostaglandins, thromboxanes, and leukotrienes. It also inhibits the migration of neutrophils to areas of inflammation, capillary permeability, edema, and the accumulation of mast cells associated with the release of histamine [48]. However, long-term administration of PDN can cause side effects such as kidney damage, depression, anxiety, dementia, and osteoporosis, which could further contribute to the deterioration of the patient with MS. Cellular experiments have also shown that silymarin can inhibit a variety of inflammation-related signaling pathways (such as the NF-κB pathway) [42]. Although there are no data on the reno-protective effect of *T. lucida*, it was observed in this work that the treatments derived from the plant, except for HN, can exert an effect leading to a reduction in extravasation in these organs (right and left kidneys). For example, it was shown that SC protects the myocardium from ischemia by inhibiting OXE and cell apoptosis [49]. 

### 3.5. Cytokines as a Measure of Neuroinflammation

For a long time, it has been observed that consuming CPZ through food induces demyelination of brain structures due to the death of oligodendrocytes. However, how this phenomenon occurs still needs to be clarified. Zirngibl et al. have proposed two possible mechanisms of action for CPZ demyelination. The first is that the damage generated in oligodendrocytes is caused by mitochondrial dysfunction or reduced myelin synthesis, a mechanism called “intrinsic cell damage” [3]. It is well known that CPZ causes mitochondrial abnormalities in mouse liver by inducing mitochondrial enlargement. This severely alters the metabolic rate as well as oxidative phosphorylation by reducing mitochondrial enzymes that contain copper as a cofactor (monoamine oxidase and cytochrome c (Cyt c) oxidase [50,51]. The second mechanism consists of damage to oligodendrocytes by inflammatory molecules resident cells in the brain, such as oligodendrocytes, astrocytes and microglia, or peripheral immune cells neutrophils or T cells; thus, this mode of action is called “Extrinsic cell damage” [3]. This second mechanism proposed by Zirngibl et al. involves a complex immune response. It has been observed that there are almost T cells present during CPZ administration, which is why it is believed that there is no involvement in the BBB [14].

Recently, Kaddatz et al. found reduced numbers of cytotoxic CD8+ T and even fewer CD4 T cells accumulated within the corpus callosum of CPZ-treated mice up to week 5 [52,53]. It has been proposed that the effect of cuprizone on demyelination in the CNS is partly due to the increase in TNF-α and its cytotoxic effect on oligodendrocyte cells [54]. Most likely, the ability of *T. lucida* to block the actions of CPZ could be associated with its immunomodulatory effect on TNF-a and IL-1b (Figure 5, panels c and d), two cytokines that are relevant in the development of MS [12].

It is known that during the development of MS, autoreactive T cells are activated in the periphery by a systemic or local factor through a mechanism of molecular mimicry (epitopes shared by the CNS autoantigen) and possibly infectious agents. These activated CD4+ T lymphocytes acquire the ability to cross the CNS by encountering an antigen-presenting cell (DC, macrophage, or microglia) that expresses MHC-II on its surface, which when stimulated, acquires a type 1 (Th1) phenotype and produces Proinflammatory ILs (TNF-α INF-γ, IL-1β, IL-2, and IL-12) and chemokines. These induce clonal proliferation of T lymphocytes and activate macrophages and microglia, producing an increase in the expression of molecules of endothelial adhesion, increasing the permeability of the BBB and allowing a high passage of mononuclear cells [11,53,55]. Macrophages and microglia secrete neurotoxic mediators such as TNF-α, nitric oxide (NO), MMPs, and ROS, contributing to myelin and axon damage [55]. Also, during the development of MS, there are type 2 helper T lymphocytes (Th2) that release anti-inflammatory ILs (IL-4, IL-5, IL10, and IL-13) that try to decrease the proinflammatory state. The results of our evaluation suggest that TlHx exerts an immunomodulatory effect by increasing the production of anti-inflammatory ILs such as IL-4 and IL-10 (Figure 5a,b). The evaluation of the anti-inflammatory effect of *T. lucida* indicated that of the two extracts evaluated, TlHx had the greatest effect, with 92.7% inhibition of edema in mouse ears, indicating a possible mechanism of action on the inhibition of COX2 [21]. The results with TlHx and TlAcOEt indicate the possible synergy of the five compounds contained in these treatments since the extracts turned out to have a larger effect, not only immunomodulatory but also in all the parameters evaluated.

## 4. Materials and Methods

### 4.1. Plant Material

*T. lucida* was acquired through local producers in the municipality of Xochitepec in Morelos, Mexico, in September 2020. One specimen was sent to the herbarium of the Ethnobotanical Garden, Cuernavaca, Morelos, Mexico (Instituto Nacional de Antropología e Historia), for taxonomic classification (INAH-2086) by the Master of Science Margarita Ávilez and Macrina Fuentes. The plant material (flowers, leaves, and stems) was kept away from light and allowed to dry in the shade for three weeks at room temperature. Subsequently, the plant was pulverized in an electric pulvex mill (Büchi R-114, Büchi Labortechnik, Flawil, Switzerland) until particles of 4 to 6 mm were obtained.

### 4.2. Preparation of Extracts

A total of 9.0 kg of plant material was used. It was first placed in a 20 L transparent glass container for three complete sequential extractions by maceration with hexane (Hx) at a concentration of 1:2 (weight/volume) for 24 h at room temperature. Afterward, it was distilled under reduced pressure in a rotary evaporator for subsequent freeze-drying. To obtain the ethyl acetate extract (TlAcOEt), we repeated the extraction procedure with AcOEt. 

### 4.3. Isolation and Identification of Five Coumarins

For the isolation and identification of the five coumarins, the *T. lucida* extracts were fractionated using the open column chromatography technique (200 × 600 mm), utilizing a column previously packed with silica gel 60 (109,385, Merck) and a gradient system Hx/ethyl acetate (AcOEt) as the mobile phase. We started with a system of 100% Hx, followed by Hx/AcOEt at proportions of 90:10, 80:20, 70:30, 60:40, 50:50, 40:60, 30:70, and 100% AcOEt. The column was finally washed with 100% MeOH. The system proportion was changed every five fractions (F) of 300 mL collected. At the end and according to their chemical profile by thin layer chromatography (TLC), the fractions were mixed and analyzed by HPLC (Waters, Milford, MA, USA). The chromatographic separation of TlHx and TlAcOEt allowed the isolation and identification of five bioactive coumarins; three of these coumarins (DF, HN, and SC) have already been reported by other authors [18,25], and two (IC and PE) were reported for the first time in 2020 [25].

### 4.4. HPLC Analysis of Extracts and Coumarins of T. lucida

The chromatographic analysis was performed using an HPLC system with a 2996 Separation module and a photodiode array detector and analyzed using the Empower 3 software. Chemical separation was achieved using a Supelcosil LC-F column (4.6 mm × 250 mm i.d., 5-µm particle size) (Sigma-Aldrich, Bellefonte, PA, USA). The mobile phase comprised a trifluoroacetic acid aqueous solution of 0.5% (solvent A) and acetonitrile (solvent B). The gradient system was as follows: 0–1 min, 0% B; 2–3 min, 5% B; 4–20 min, 30% B; 21–23 min, 50% B; 14–15 min; 24–25 min, 80% B; 26–27 min, 100% B; and 28–30 min, 0% B. The flow rate was maintained at 0.9 mL min^−1^, and the sample injection volume was 10 µL. Absorbance was measured at 330 nm. Compared with the standards, the compounds were identified by their UV spectra and retention times (RT). After the identification of the compounds, standardization of the extracts was carried out for their coumarin content, for which a standard curve of each coumarin was built with an initial concentration of 40 µg/mL followed by 20, 10, 5, 2.5, 1.25, 0.625, 0.312 and 0.156. µg/mL. Each sample was injected into the HPLC, and the chromatograms obtained were observed at a wavelength of 330 nm. The AUC of the peaks obtained was integrated to generate the equation y = mx + b, considering R2 = 0.99 to 1 as adequate. The results obtained determined the concentration in µg/mg of extract by coumarin [32].

### 4.5. Animals

Male BALB/c mice (10-week-old) obtained from our animal facilities were used. Eleven groups of fifteen mice each were housed and maintained under pathogen-free conditions in the animal facility at the Southern Biomedical Research Center of the Mexican Institute of Social Security (IMSS) under constant temperature (21–23 °C) and humidity (45–50%) conditions, with a 12 h light/dark cycle and free access to water and standard food. All the experiments followed the guidelines in the National Institutes of Health Guide for the Care and Use of Laboratory Animals, and experimental protocols were reviewed. For the euthanasia of the mice, the overdose technique with sodium pentobarbital was selected, which is accepted by the official Mexican Norm with the number NOM-062-ZOO-1999 (Technical specifications for the production, care, and use of animals from the laboratory). Furthermore, the experimental protocol was authorized by the local Health Research Committee IMSS, with approval number: R-2020-1702-073.

### 4.6. Model of Deteriorous Behavior, Motor Damage and Neuroinflamation Induced with Cuprizone

For five weeks, motor damage was induced through the forced administration of CPZ (Sigma Aldrich- C9012) 40 mg/kg (VEH, damage group) using 1% tween 20 as a diluent. Eleven groups were formed.

1. Healthy mice were treated with Tween 20 at 1% only. The remaining groups were treated with CPZ (Bis(cyclohexanone)oxaldihydrazone, daily for five weeks), either alone or co-administered at the end of week 2 with the respective treatments by the oral pathway.
2. Damage group (VEH, Tween 20 at 1%).
3. Sylimarin (SLM, 200 mg/kg).
4. Prednisone (PDN, 2 mg/kg).

There were seven treatments based on *T. lucida.*

5. Isoprenyloxycumarin (IC, 25 mg/kg).
6. Herniarin (HN, 25 mg/kg).
7. 7-O-prenyl-scopoletin (PE, 25 mg/kg).
8. Dimethylfraxetin (DF, 25 mg/kg).
9. Scoporone (SC, 25 mg/kg).
10. TlHx (25 mg/kg).
11. TlAcOEt (25 mg/kg).

Furthermore, all treatments were diluted in Tween 20 at 1% and administered by oral pathway for 3 weeks. Every third day, from the first administration, the Irwin test observations performed. At the end of each week, the “rota rod” (motor skills) test was performed, and at the end of week 5, the open field evaluation test. After this, the animals were sacrificed to obtain the target organs.

### 4.7. Behavioral Activity

#### 4.7.1. The Irwin Test

This test was performed at three points in the procedure: (1) when there was no disturbance of the cage or handling of the animal, (2) during handling of the animal and administration of CPZ or the treatment, and (3) in the first 15 min after administration. The animals in each group were evaluated for the presence or absence of any behavioral symptoms or physiological manifestations three days per week; the parameters evaluated are shown in Table 4 [33].

#### 4.7.2. Rota-Rod Test

Motor coordination was evaluated using the rota-rod test, performed at the end of each week. The rota-rod device comprised a 3 cm diameter rotating rod divided into five 6 cm wide tracks, a time detector that stopped when the mouse landed on the track, and software that recorded the time spent on the rod. For each animal, the time spent on the rod was evaluated for 5 min. Each minute, the speed increased, i.e., the first minute = 4 RPM, minute two = 12 RPM, minute three = 20 RPM, minute four = 28 RPM, and minute five = 36 RPM. The results were calculated by integrating the AUC of the five experimental weeks.

#### 4.7.3. Open Field Test (OFT)

The open field test was used to evaluate the exploratory activity of the animal. The OFT comprised an acrylic box (transparent walls and black floor, 30 × 30 × 15 cm) divided into nine squares of equal area. Each mouse was placed in the box, and the behavior was videotaped for 5 min under red light for analysis. The parameters measured were the number of times that the animal crossed the quadrats posing on all four legs, which was classified as total number of crossings (TC), the number of crossings to the periphery (CP), and the number of crossings to the center (CC). The number of rearing (R) and grooming (G) behaviors was also observed, as was the number of stools. The test was performed at the end of week 5.

### 4.8. Measure of Vascular Permeability with Extravasation of Evans Blue Test

To measure vascular permeability in mice with motor damage induced with CPZ, an intravenous injection of Evans blue (EB) was performed. A sterile 0.5% EB solution was prepared in PBS (the solution was filter-sterilized to remove undissolved particles), and 200 µL was injected into the tail vein of the mouse. After 1 h, the mice were sacrificed to obtain the brain, spleen, and right and left kidneys. The organs of interest were obtained, weighed, and collocated in 500 µL of formamide. All tubes containing the samples were placed in a water bath at 55 °C and incubated for 24 h to extract EB from the tissue. After that time, the tubes were centrifuged (10 min, at 14,000 RPM) to sediment the remaining tissue fragments. The absorbance was measured at 630 nm, using 500 µL of formamide as a blank. Data were calculated using the EB extinction coefficient ε = 7.81 × 10^4^ [56].

### 4.9. Cytokine Quantification by ELISA

The brains were macerated in a frozen mortar with ice-cold PBS-PMSF (0.1%) 1:5 *w*/*v*. The suspensions were centrifuged at 14,000 RPM for 7 min and the supernatants were used to determine the concentration of cytokines by ELISA, following the manufacturer’s instructions. Mouse IL-1β, IL-4, IL-10, and TNFα ELISA kits were purchased from OptEIA™ BD. 

Briefly, 96-well flat-bottomed ELISA plates were coated with the respective capture antibody and incubated overnight at 4 °C. Non-specific binding sites were blocked by incubating for 30 min at RT with PBS-5% fetal bovine serum (FBS). Next, the sample was added and incubated for 2 h at room temperature. Then, the plates were incubated with the corresponding detection anti-cytokine-HRP antibodies for 30 min at room temperature. Bound complexes were detected by reaction with tetramethylbenzidine substrate after 30 min incubation in the dark. The reaction was stopped with H_2_SO_4_ 2 N and the absorbance was measured at 490 nm at 37 °C in a VERSAmax ELISA plate reader (Molecular Devices). The cytokine concentration was calculated according to standard curves for each cytokine and reported as pg/mg protein.

### 4.10. Statistics

The data were analyzed using InStat (GraphPad, San Diego, CA, USA) and expressed as mean ± SD. One-way ANOVA was used to compare groups or treatments with a Tukey test for Rr and quantification of ILs. Dunnet Test was used to analyze OFT, and a student t-test for extravasation of EB. The Chi-square test from Excel was used for the relativized behavior results in the Irwin test. Differences were considered significant when * *p* < 0.05, compared with the VEH group, for all assays.

## 5. Conclusions

The results presented here allow us to conclude that the extracts and five coumarins (dimethylfraxetine, 7-O-prenylscopoletin, 7-isoprenyloxycoumarin, herniarin, and scoporone) from *T. lucida* may have pharmacological effects for controlling motor impairment, behavioral impairment and systemic inflammation. At the level of the central nervous system, they can provide protection against tissue damage caused by increased vascular permeability, mainly due to protection of the BBB, in mice with experimental encephalomyelitis induced with CPZ.

## Figures and Tables

**Table 1:** Concentrations of five coumarins isolated from *T. lucida*, in two extracts.

Extract	IC	HN	DF	SC	PE
	(µg/mg)	(µg/mg)	(µg/mg)	(µg/mg)	(µg/mg)
TlHx	40.1	79.9	19.9	15,528.4	0.1
TlAcOEt	18.1	381.9	75.3	230,692.7	3.05

7-Isoprenyloxycoumarin (IC), herniarin (HN), Dimethylfraxetine (DF), Scoporone (SC) and 7-O-prenylscopoletin (PE).

**Table 2:** Relative piloerection (%) observed during the Irwin test.

Week	Healtly	VEH	SLM	PDN	TlHx	TlAcOEt	DF	PE	IC	HN	SC
		Cuprizone									
1	N/O	N/O	N/O	N/O	N/O	N/O	N/O	N/O	N/O	N/O	N/O
2	N/O	20 *	13 *	15 *	27 *	20 *	7 *	N/O	13 *	20 *	27 *
3	N/O	53 *	40 *	37 *	27 *	47 *	27 *	40 *	33 *	33 *	20 *
4	N/O	93 *	73 *	40 *	27 *	33 *	13 *	33 *	40 *	27 *	13 *
5	N/O	93 *	60 *	26 *	20 *	20 *	20 *	7 *	13 *	20 *	6 *

Vehicle (VEH), silemarine, (SLM), Prednisone (PDN), dimethylfraxetine (DF), 7-O-prenylscopoletin (PE), 7-Isoprenyloxycoumarin (IC), herniarin (HN), and scoporone (SC). N/O = no observed. Data were analyzed with χ_2_ test (n = 15). * indicates a significant difference at *p* < 0.05, comparing all treatments with respect to time in weeks of exposure to cuprizone.

**Table 3:** Relative crowding behavior (%) of mice exposed to different treatments during the Irwin test.

Week	Healtly	VEH	SLM	PDN	TlHx	TlAcOEt	DF	PE	IC	HN	SC
		Cuprizone									
1	N/O	27 *	33 *	20 *	20 *	13 *	27 *	13 *	40 *	33 *	20 *
2	N/O	33 *	33 *	37 *	27 *	27 *	40 *	60 *	47 *	47 *	40 *
3	N/O	87 *	53 *	40 *	27 *	40 *	33 *	53 *	33 *	40 *	33 *
4	N/O	87 *	33 *	28 *	20 *	33 *	20 *	40 *	27 *	27 *	20 *
5	N/O	100 *	20 *	20 *	13 *	N/O	N/O	N/O	13 *	13 *	13 *

Vehicle (VEH), silemarine, (SLM), Prednisone (PDN), dimethylfraxetine (DF), 7-O-prenylscopoletin (PE), 7-Isoprenyloxycoumarin (IC), herniarin (HN), and scoporone (SC). N/O = no observed. Data were analyzed with χ_2_ test (n = 15). * indicates a significant difference when *p* < 0.05, comparing all treatments with respect to time in weeks of exposure to cuprizone.

**Table 4:** Parameters observed during the Irwin Test.

Undisturbed Observations		Animal Handling		After Administration	
a	Writhing	f	Abdominal tone	n	Crowding
b	Jumping	g	Limb tone	o	Ataxia
c	Tremor	h	Pupil size	p	Increased locomotion
d	Convulsion	i	Visual placement	q	No exploration
e	Raised hair	j	Lacrimation		
		k	Salivation		
		m	Diarrhea		

## Data Availability

No electronic file with the data supporting the results presented is available, but the data are included in specific attachments as Supplementary Data. Samples of the compounds: then 7-Isoprenyloxycumarin (IC) is more concentrated in the less polar extract (TlHx). At the same time, Herniarin (HN)), Dimethylfraxetin (DF), Scoporone (SC), and 7-O-prenylscopoletin (PE), are available from the authors.

## Supplementary Materials

The following supporting information can be downloaded at: https://www.mdpi.com/article/10.3390/ph16101391/s1, Data S1: NMR of IC, Data S2: NRM of HN, Data S3: NRM of PE, Data S4: NRM of DF, and Data S5: NRM of SC. Figure S1: Standardization chromatogram and UV spectra of IC; Figure S2: Standardization chromatogram and UV spectra of HN; Figure S3: Standardization chromatogram and UV spectra of PE, Figure S4: Standardization chromatogram and UV spectra of DF; and Figure S5: Standardization chromatogram and UV spectra of SC. Table S1: ANOVA test of OFT; Table S2: ANOVA test of Rr; Table S3: ANOVA test of EB; and Table S4: ANOVA test of ILs.

## References

[1] Peng Y., Zhu F.Z., Chen Z.X., Zhou J.X., Gan L., Yang S.S., Gao S., Liu Q.Q. (2019). Characterization of myelin oligodendrocyte glycoprotein (MOG)35-55-specific CD8+ T cells in experimental autoimmune encephalomyelitis. Chin. Med. J..

[2] Baecher-allan C., Kaskow B.J., Weiner H.L. (2018). Review Multiple Sclerosis: Mechanisms and Immunotherapy. Neuron.

[3] Zirngibl M., Assinck P., Sizov A., Caprariello A.V., Plemel J.R. (2022). Oligodendrocyte death and myelin loss in the cuprizone model: An updated overview of the intrinsic and extrinsic causes of cuprizone demyelination. Mol. Neurodegener..

[4] Castillo-Álvarez F., Marzo-Sola M.E. (2017). Role of intestinal microbiota in the development of multiple sclerosis. Neurología.

[5] Terry R.L., Ifergan I., Miller S.D. (2016). Experimental Autoimmune Encephalomyelitis in Mice. Methods Mol. Biol..

[6] LaMothe R.A., Kolte P.N., Vo T., Ferrari J.D., Gelsinger T.C., Wong J., Chan V.T., Ahmed S., Srinivasan A., Deitemeyer P. (2018). Tolerogenic nanoparticles induce antigen-specific regulatory T cells and provide therapeutic efficacy and transferrable tolerance against experimental autoimmune encephalomyelitis. Front. Immunol..

[7] Macías-Cosme K., Cervantes-Llanos M., Marín-Prida J., Falcón-Cama V., Pentón-Arias E., Pentón-Rol G. (2012). Generation of a murine chronic progressive experimental autoimmune encephalomyelitis model for molecular pharmacology studies in multiple sclerosis. Biotecnol. Apl..

[8] Constantinescu C.S., Farooqi N., O’Brien K., Gran B. (2011). Experimental autoimmune encephalomyelitis (EAE) as a model for multiple sclerosis (MS). Br. J. Pharmacol..

[9] Blaschek A., Karenfort M., Schimmel M., Pritsch M., Storm K., Gravesande V., Weber M., Schmoeger M., Seidl R., Prayer D. (2018). Clinical and magnetic resonance imaging features of children, adolescents, and adults with a clinically isolated syndrome. SC. Eur. J. Paediatr. Neurol..

[10] García-Díaz B., Estivill-Torrús G. (2008). Modelos de experimentación animal para la investigación en esclerosis múltiple. Rev. Española Escler. Mult..

[11] Wootla B., Eriguchi M., Rodriguez M. (2012). Is multiple sclerosis an autoimmune disease?. Autoimmune Dis..

[12] Barthelmes J., Tafferner N., Kurz J., de Bruin N., Parnham M.J., Geisslinger G., Schiffmann S. (2016). Induction of experimental autoimmune encephalomyelitis in mice and evaluation of the disease-dependent distribution of immune cells in various tissues. J. Vis. Exp..

[13] Torre-Fuentes L., Moreno-Jiménez L., Pytel V., Matías-Guiu J.A., Gómez-Pinedo U., Matías-Guiu J. (2019). Experimental models of demyelination and remyelination. Neurología.

[14] Zhan J., Mann T., Joost S., Behrangi N., Frank M., Kipp M. (2020). The Cuprizone Model: Dos and Do Nots. Cells.

[15] Gudi V., Gingele S., Skripuletz T., Stangel M. (2014). Glial response during cuprizone-induced de- and remyelination in the CNS: Lessons learned. Front. Cell. Neurosci..

[16] Torkildsen Ø., Brunborg L.A., Myhr K.M., Bø L. (2008). The cuprizone model for demyelination. Acta Neurol. Scand..

[17] González-Trujano M.E., Gutiérrez-Valentino C., Hernández-Arámburo M.Y., Díaz-Reval M.I., Pellicer F. (2019). Identification of some bioactive metabolites and inhibitory receptors in the antinociceptive activity of Tagetes lucida Cav. Life Sci..

[18] Pérez-Ortega G., González-Trujano M.E., Ángeles-López G.E., Brindis F., Vibrans H., Reyes-Chilpa R. (2016). Tagetes lucida Cav.: Ethnobotany, phytochemistry and pharmacology of its tranquilizing properties. J. Ethnopharmacol..

[19] Capunzo M., Brunetti L., Cavallo P., Boccia G., Caro F.D.E., Ieluzzi M. (2003). Antimicrobial activity of dry extracts of Tagetes lucida from Guatemala. J. Prev. Mesicine Hyg..

[20] Adams M., Gmünder F., Hamburger M. (2007). Plants traditionally used in age related brain disorders-A survey of ethnobotanical literature. J. Ethnopharmacol..

[21] Guadarrama-Cruz G., Alarcon-Aguilar F.J., Lezama-Velasco R., Vazquez-Palacios G., Bonilla-Jaime H. (2008). Antidepressant-like effects of Tagetes lucida Cav. in the forced swimming test. J. Ethnopharmacol..

[22] Guzmán Gutiérrez S.L.G., Reyes Chilpa R.R., Bonilla Jaime H. (2014). Medicinal plants for the treatment of “nervios”, anxiety, and depression in Mexican Traditional Medicine. Rev. Bras. Farmacogn..

[23] Bonilla-Jaime H., Guadarrama-Cruz G., Alarcon-Aguilar F.J., Limón-Morales O., Vazquez-Palacios G. (2015). Antidepressant-like activity of *Tagetes lucida* Cav. is mediated by 5-HT1A and 5-HT2A receptors. J. Nat. Med..

[24] Malik A., Kushnoor A., Saini V., Singhal S., Kumar S., Yadav Y.C. (2011). In vitro antioxidant properties of Scopoletin. J. Chem. Pharm. Res..

[25] Monterrosas-brisson N., Herrera-ruiz M., Jiménez-ferrer E., Bahena-pérez R., Avilés-flores M., Fuentes-mata M., Martínez-duncker I., González-cortazar M., Avilés-flores M., Fuentes-mata M. (2020). Anti-inflammatory activity of coumarins isolated from Tagetes lucida Cav. Nat. Prod. Res..

[26] Sandra Liliana P.D., Manasés G.C., Enrique J.F., Rubén R.R., Cinthya B.P., Belen M.H.G., Alejandro Z., Maribel H.R. (2022). Isolation, chemical characterization, and anti-inflammatory activity of coumarins, flavonoids, and terpenes from *Tagetes lucida*. Nat. Prod. Res..

[27] Porras-Dávila S.L., Jiménez-Ferrer E., Román Ramos R., González-Cortazar M., Almanza-Pérez J.C., Herrera-Ruiz M. (2022). Herniarin, Dimethylfraxetin and Extracts from Tagetes lucida, inPsychosis Secondary to Ketamine and Its Interaction with Haloperidol. Plants.

[28] Liliana Porras-Dávila S., Zamilpa A., Jiménez-Ferrer E., Jiménez-Aparicio A., Alejandra Santillan-Urquiza M., Díaz-Patricio F., Herrera-Ruiz M. (2023). Anti-Inflammatory and Neuroprotective Effects of Standardized Fractions in Herniarin and Daphnoretin from Distictis buccinatoria. Chem. Biodivers.

[29] Hernández T., Canales M., Flores C., García A.M., Durán A., Avila J.G. (2006). Antimicrobial activity of *Tagetes lucida*. Pharm Biol..

[30] Chaves O.S., Teles Y.C.F., De Oliveira Monteiro M.M., Mendes L.D.G., De Fátima Agra M., De Andrade Braga V., Silva T.M.S., de Fátima Vanderlei de Souza M. (2017). Alkaloids and phenolic compounds from *Sida rhombifolia* L. (Malvaceae) and vasorelaxant activity of two indoquinoline alkaloids. Molecules.

[31] Bubols G.B., Vianna D.d.R., Medina-Remón A., von Poser G., Lamuela-Raventos R.M., Eifler-Lima V.L., Garcia S.C. (2013). The Antioxidant Activity of Coumarins and Flavonoids. Mini-Rev. Med. Chem..

[32] Santibáñez A., Herrera-Ruiz M., González-Cortazar M., Nicasio-Torres P., Sharma A., Jiménez-Ferrer E. (2022). Pharmacokinetics and Tissue Distribution of Coumarins from Tagetes lucida in an LPS-Induced Neuroinflammation Model. Plants.

[33] Kopanitsa M.V., Lehtimäki K.K., Forsman M., Suhonen A., Koponen J., Piiponniemi T.O., Kärkkäinen A.M., Pavlidi P., Shatillo A., Sweeney P.J. (2021). Cognitive disturbances in the cuprizone model of multiple sclerosis. Genes Brain Behav..

[34] Mathiasen J.R., Moser V.C. (2018). The Irwin Test and Functional Observational Battery (FOB) for Assessing the Effects of Compounds on Behavior, Physiology, and Safety Pharmacology in Rodents. Curr. Protoc. Pharmacol..

[35] McPhetres J., Zickfeld J.H. (2022). The physiological study of emotional piloerection: A systematic review and guide for future research. Int. J. Psychophysiol..

[36] Sternberg Z. (2012). Sympathetic Nervous System Dysfunction in Multiple Sclerosis, Linking Neurodegeneration to a Reduced Response to Therapy. Curr. Pharm. Des..

[37] Andrade J.C., Monteiro Á.B., Andrade H.H.N., Gonzaga T.K.S.N., Silva P.R., Alves D.N., Castro R.D., Maia M.S., Scotti M.T., Sousa D.P. (2021). Involvement of GABA A Receptors in the Anxiolytic-Like Effect of Hydroxycitronellal. BioMed Res. Int..

[38] Kraeuter A.K., Guest P.C., Sarnyai Z. (2019). The Open Field Test for Measuring Locomotor Activity and Anxiety-like Behavior. Methods Mol. Biol..

[39] Liu H., Huang X., Xu J., Mao H., Li Y., Ren K., Ma G., Xue Q., Tao H., Wu S. (2021). Dissection of the relationship between anxiety and stereotyped self-grooming using the Shank3B mutant autistic model, acute stress model and chronic pain model. Neurobiol. Stress.

[40] Wang X., Chang L., Wan X., Tan Y., Qu Y., Shan J., Yang Y., Ma L., Hashimoto K. (2022). (R)-ketamine ameliorates demyelination and facilitates remyelination in cuprizone-treated mice: A role of gut–microbiota–brain axis. Neurobiol. Dis..

[41] Franco-Pons N., Torrente M., Colomina M.T., Vilella E. (2007). Behavioral deficits in the cuprizone-induced murine model of demyelination/remyelination. Toxicol. Lett..

[42] Guo H., Cao H., Cui X., Zheng W., Wang S., Yu J., Chen Z. (2019). Silymarin’s inhibition and treatment effects for Alzheimer’s disease. Molecules.

[43] Kren V., Walterová D. (2005). Silybin and silymarin--new effects and applications. Biomed. Pap. Med. Fac. Univ. Palacky. Olomouc. Czech. Repub..

[44] Berghoff S.A., Düking T., Spieth L., Winchenbach J., Stumpf S.K., Gerndt N., Kusch K., Ruhwedel T., Möbius W., Saher G. (2017). Blood-brain barrier hyperpermeability precedes demyelination in the cuprizone model. Acta Neuropathol. Commun..

[45] Abbott N.J., Patabendige A.A.K., Dolman D.E.M., Yusof S.R., Begley D.J. (2010). Structure and function of the blood-brain barrier. Neurobiol. Dis..

[46] Radu M., Chernoff J. (2013). An in vivo assay to test blood vessel permeability. J. Vis. Exp..

[47] Goldim M.P.d.S., Della Giustina A., Petronilho F. (2019). Using Evans Blue Dye to Determine Blood-Brain Barrier Integrity in Rodents. Curr. Protoc. Immunol..

[48] Krasselt M., Baerwald C. (2016). Efficacy and safety of modified-release prednisone in patients with rheumatoid arthritis. Drug Des. Devel. Ther..

[49] Wan C., Wei Y., Ma J., Geng X. (2018). Protective effects of scoparone against ischemia-reperfusion-induced myocardial injury. Mol. Med. Rep..

[50] Suzuki K. (1969). Giant hepatic mitochondria: Production in mice fed with cuprizone. Science.

[51] Kesterson J.W., Carlton W.W. (1971). Monoamine oxidase inhibition and the activity of other oxidative enzymes in the brains of mice fed cuprizone. Toxicol. Appl. Pharmacol..

[52] Kaddatz H., Joost S., Nedelcu J., Chrzanowski U., Schmitz C., Gingele S., Gudi V., Stangel M., Zhan J., Santrau E. (2021). Cuprizone-induced demyelination triggers a CD8-pronounced T cell recruitment. Glia.

[53] Slavin A., Ewing C., Liu J., Ichikawa M., Slavin J., Bernard C.C.A. (1998). Induction of a multiple sclerosis-like disease in mice with an immunodominant epitope of myelin oligodendrocyte glycoprotein. Autoimmunity.

[54] Pasquini L.A., Calatayud C.A., Bertone Uña A.L., Millet V., Pasquini J.M., Soto E.F. (2007). The neurotoxic effect of cuprizone on oligodendrocytes depends on the presence of pro-inflammatory cytokines secreted by microglia. Neurochem. Res..

[55] Miller S.D., Karpus W.J., Davidson T.S. (2010). Experimental Autoimmune Encephalomyelitis in the Mouse. Curr. Protoc. Immunol..

[56] Honeycutt S.E., O’Brien L.L. (2020). Injection of Evans blue dye to fluorescently label and image intact vasculature. Biotechniques.

