# Supplementary material for: Effects of Five Coumarins and Standardized Extracts from Tagetes lucida Cav. on Motor Impairment and Neuroinflammation Induced with Cuprizone
Source: Pharmaceuticals (Basel). 2023 Sep 30;16(10):1391. doi: 10.3390/ph16101391 (PMC10610053; doi:10.3390/ph16101391)
Supplement: Supplementary file 1 [file pharmaceuticals-16-01391-s001.zip › pharmaceuticals-2610056-supplementary.pdf]

Effect of five coumarins and standardized extracts from *Tagetes lucida* on motor impairment and neuroinflammation induced with cuprizone.

Gabriela Castro-Martínez [1,2], Maribel Herrera-Ruiz [2], Manases González-Cortázar [2], Sandra Liliana Porras -Dávila [2], Julio Cesar Almanza-Pérez [3], Enrique Jiménez-Ferrer [2]*

[1] Doctorado en Ciencias Biológicas y de la Salud, Universidad Autónoma Metropolitana, Ciudad de México, México. gcm_19@hotmail.com

[2] Centro de Investigación Biomédica del Sur, Instituto Mexicano Del Seguro Social, Argentina No. 1, Col. Centro, Xochitepec, Morelos 62790, México. gcm_19@hotmail.com; cibis_herj@yahoo.com.mx; gmanases2000@gmail.com; davilasp1117@gmail.com; jimenezferrer_mx@yahoo.com.

[3] Departamento de Ciencias de la Salud, División de Ciencias Biológicas y de la Salud, Universidad Autónoma Metropolitana-Iztapalapa, Av. Ferrocarril San Rafael Atlixco 186, Ciudad de México, México jcap@xanum.uam.mx

* Correspondence: E-J-F jesus.jimenezf@imss.gob.mx; enriqueferrer_mx@yahoo.com

Identification of coumarins

7-Isoprenyloxycoumarin (IC)

Data S1; $C_{14}H_{14}O_3$: ^{1}H NMR (400 MHz, Chloroform-d): δ 6.14 (1H, d, 9.3 Hz, H-3), 7.58 (1H, d, 9.7 Hz, H-4), 7.29 (1H, d, 8.5 Hz, H-5), 6.74 (1H, dd, 2.3, 8.5 Hz, H6), 6.67 (1H, d, 2.3 Hz, H-8), 4.48 (2H, br, d, 6.6 Hz, H-1'a and H-1'b), 5.37 (1H, dd, 6.6, 6.6 Hz, H-2'), 1.71 (3H, s, H-4'), 1.68 (3H, s, H-5'); ^{13}C NMR (100 MHz, Chloroform-d): δ 160.76 (C-2), 112.27 (C-3), 143.16 (C4), 128.40 (C-5), 112.27 (C-6), 161.62 (C-7), 100.99 (C-8), 155.28 (C-9), 111.95 (C-10), 64.94 (C-1′), 118.31 (C-2′), 138.54 (C-3′), 25.34 (C-4′), 17.80 (C-5′).

Herniarin (HN)

Data S2; $C_{10}H_8O_3$: ^{1}H NMR (600 MHz, Chloroform-d): δ 6.24 (1H, d, 9.0 Hz, H-3), 7.63 (1H, d, 9.7 Hz, H-4), 7.37 (1H, d, 9.0 Hz, H-5), 6.83 (1H, dd, 2.0, 8.3 Hz, H-6), 6.80 (1H, d, 2.7 Hz, H-8), 3.87(3H, 7-OCH_3); ^{13}C NMR (100 MHz, Chloroform-d): δ 161.12 (C-2), 113.04 (C2), 143.36 (C-4), 128.7 (C-5), 112.52 (C-6), 162.79 (C-7), 100.80 (C-8), 155.86 (C-9), 112.48 (C-10), 55.86 (- OCH_3).

7-O-prenylscopoletin (PE)

Data S3; $C_{15}H_{15}O_4$: ^{1}H NMR (600 MHz, Chloroform-d): δ 6.24 (1H, d, 9.5 Hz, H-3), 7.60 (1H, d, 9.5 Hz, H-4), 6.84 (1H, s, H-5), 6.81 (1H, s, H-8), 4.64 (2H, d, 6.6 Hz, H-1'a and H-1'b), 5.47 (1H, dd, 6.6, 7.0 Hz, H-2'), 1.77 (3H, s, H-4'), 1.76 (3H, s, H-5'), 3.88 (3H, -OCH_3); ^{13}C NMR (100 MHz, Chloroform-d): δ 161.40 (C-2), 113.19 (C-3), 143.26 (C-4), 108.12 (C-5), 146.62 (C-6), 152.11 (C-7), 101.09 (C-8), 149.85 (C-9), 111.25 (C-10), 66.18 (C-1′), 118.53 (C-2′), 138.86 (C-3′), 25.71 (C4′), 18.24 (C-5′), 56.29 (OCH_3).

Dimethylfraxetin (DF)

Data S4; $C_{12}H_{12}O_5$: ^{1}H NMR (600 MHz, Chloroform-d): δ 6.32 (1H, d, 9.1 Hz, H-3), 7.60 (1H, d, 9.5 Hz, H-4), 6.67 (1H, s, H-5), 3.89 (3H, s, 6-OCH_3), 3.98 (3H, s, 7- OCH_3), 4.02 (3H, s, 8-OCH_3); ^{13}C NMR (100

MHz, Chloroform-d): δ 160.39 (C-2), 114.3 (C-2), 143.38 (C-4), 103.8 (C-5), 150.11 (C-6), 145.96 (C-7), 141.14 (C-8), 143.03 (C-9), 115.12 (C-10), 56.31(6-OCH_3), 61.44 (7- OCH_3), 61.77 (8-OCH_3).

Scoparone (SC)

Data S5; $C_{11}H_{10}O_4$: ^{1}H NMR (600 MHz, Chloroform-d): δ 6.24 (1H, d, 9.5 Hz, H-3), 7.59 (1H, d, 9.5 Hz, H-4), 6.84 (1H, s, H-5), 6.80 (1H, s, H-8), 3.88 (3H, s, 6- OCH_3), 3.91 (3H, s, 7-OCH_3); ^{13}C NMR (100 MHz, Chloroform-d): δ 161.26 (C-2), 113.38 (C-2), 143.22 (C-4), 108.07 (C-5), 146.3 (C-6), 152.83 (C-7), 99.83 (C-8), 149.94 (C-9), 111.37 (C-10), 56.28(6-OCH_3), 56.26 (7- OCH_3).

Standardization chromatograms

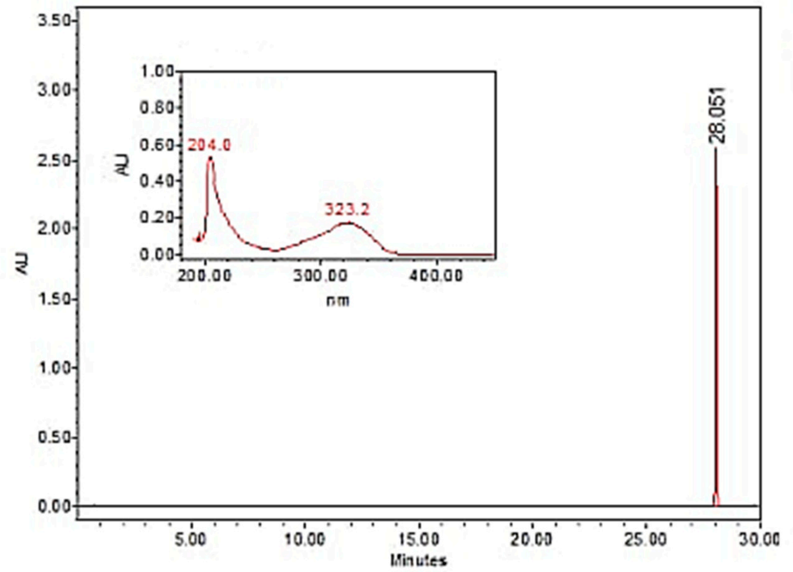

Figure S1: Standardization chromatogram and uv spectra of IC.

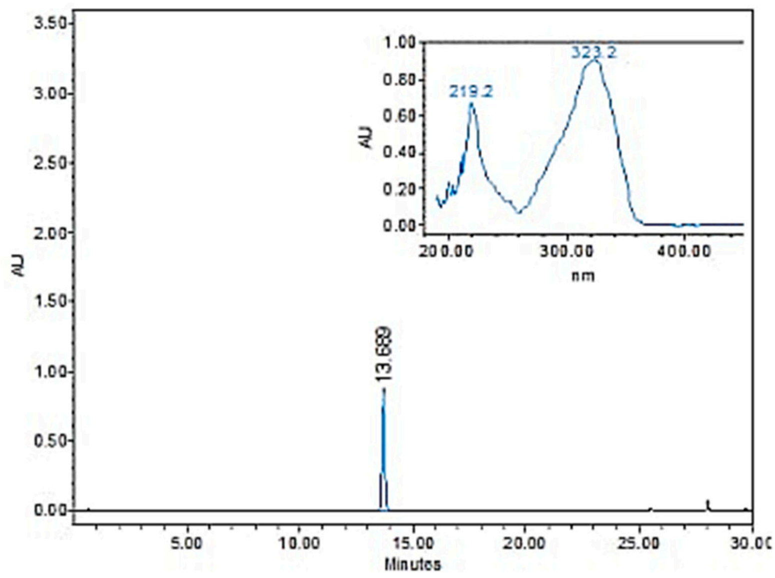

Figure S2: Standardization chromatogram and uv spectra of HN.

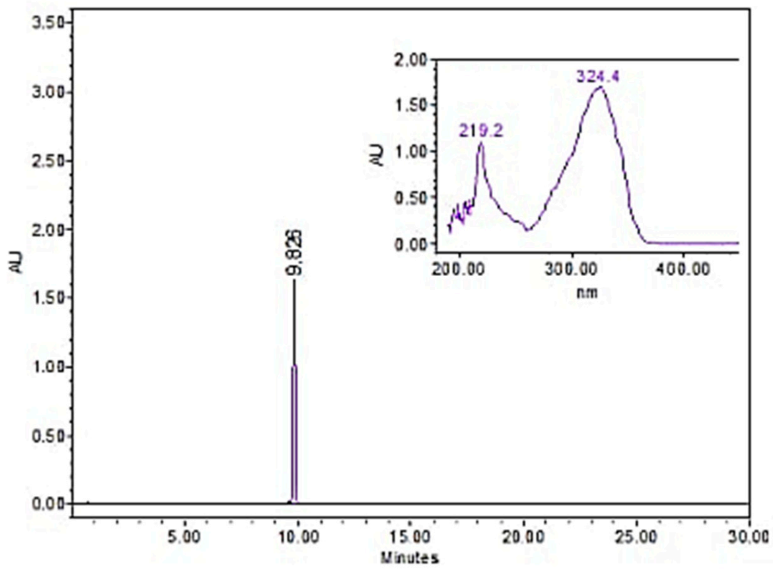

Figure S3: Standardization chromatogram and uv spectra of PE

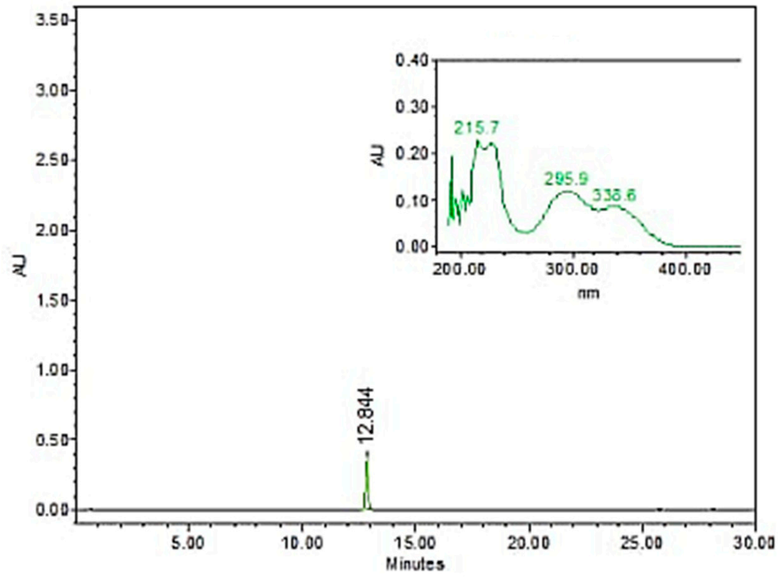

Figure S4: Standardization chromatogram and uv spectra of DF

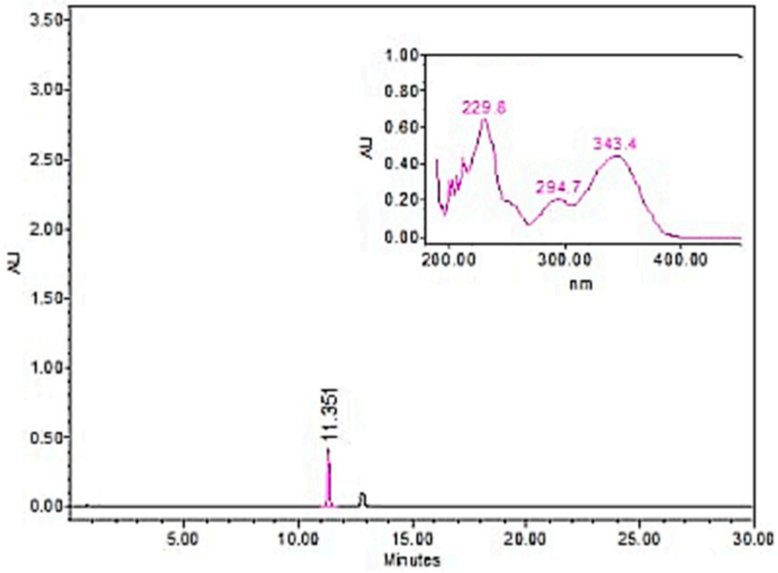

Figure S5: Standardization chromatogram and uv spectra of SC.

Statistical análisis

Table S1: ANOVA test of OFT

Table Analyzed	croosing to the periphery number	Center croosing number	Total croosing number	Rearing	Number of grooming	Stool number
**ANOVA summary**						
**F**	**16.7**	**10.7**	**16.72**	**49.16**	**57.26**	**7.627**
**P value**	**<0.0001**	**<0.0001**	**<0.0001**	**<0.0001**	**<0.0001**	**<0.0001**
**P value summary**	****	****	****	****	****	****
**Significant diff. among means (P < 0.05)?**	**Yes**	**Yes**	**Yes**	**Yes**	**Yes**	**Yes**
**R squared**	**0.6845**	**0.5815**	**0.6847**	**0.8646**	**0.8815**	**0.4976**
**Brown-Forsythe test**						
**F (DFn, DFd)**	2.638 (10, 77)	1.209 (10, 77)	3.300 (10, 77)	1.628 (10, 77)	0.4607 (10, 77)	0.09070 (10, 77)
**P value**	0.0081	0.2987	0.0013	0.1145	0.9101	0.9999
**P value summary**	**	ns	**	ns	ns	ns
**Are SDs significantly different (P < 0.05)?**	Yes	No	Yes	No	No	No
**Bartlett's test**						
**Bartlett's statistic (corrected)**	21.93	7.955	32.33	12.31	7.339	3.625
**P value**	0.0154	0.6332	0.0004	0.2646	0.6931	0.9627
**P value summary**	*	ns	***	ns	ns	ns
**Are SDs significantly different (P < 0.05)?**	Yes	No	Yes	No	No	No

Table S2: ANOVA test of Rr

Table Analyzed	36 RPM	28 RPM	20 RPM
**ANOVA summary**			
**F**	**25.06**	**43.27**	**28.42**
**P value**	**<0.0001**	**<0.0001**	**<0.0001**
**P value summary**	****	****	****
**Significant diff. among means (P < 0.05)?**	**Yes**	**Yes**	**Yes**
**R squared**	**0.6193**	**0.7375**	**0.6486**
**Brown-Forsythe test**			
**F (DFn, DFd)**	0.9986 (10, 154)	1.603 (10, 154)	0.8751 (10, 154)
**P value**	0.4472	0.1103	0.5579
**P value summary**	ns	ns	ns
**Are SDs significantly different (P < 0.05)?**	No	No	No
**Bartlett's test**			
**Bartlett's statistic (corrected)**	15.02	25.03	17.04
**P value**	0.1313	0.0053	0.0735
**P value summary**	ns	**	ns
**Are SDs significantly different (P < 0.05)?**	No	Yes	No

Table S3: ANOVA test of EB

Table Analyzed	Evans Blue Brain	Evans Blue Left Kidney	Evans Blue Right Kidney	Evans Blue Spleen
**ANOVA summary**				
**F**	**105**	**160.8**	**112.4**	**74.55**
**P value**	**<0.0001**	**<0.0001**	**<0.0001**	**<0.0001**
**P value summary**	****	****	****	****
**Significant diff. among means (P < 0.05)?**	**Yes**	**Yes**	**Yes**	**Yes**
**R squared**	**0.9317**	**0.9543**	**0.9359**	**0.9064**
**Brown-Forsythe test**				
**F (DFn, DFd)**	2.691 (10, 77)	1.091 (10, 77)	0.5111 (10, 77)	0.8006 (10, 77)
**P value**	0.007	0.3798	0.8773	0.6284
**P value summary**	**	ns	ns	ns
**Are SDs significantly different (P < 0.05)?**	Yes	No	No	No
**Bartlett's test**				
**Bartlett's statistic (corrected)**	23.78	13.83	7.087	8.726
**P value**	0.0082	0.181	0.7172	0.5583
**P value summary**	**	ns	ns	ns
**Are SDs significantly different (P < 0.05)?**	Yes	No	No	No

Table S4: ANOVA test of ILs.

Table Analyzed	IL-4	IL-10	IL-1β	TNF-α
**ANOVA summary**				
**F**	10.42	7.128	76.91	15.33
**P value**	<0.0001	<0.0001	<0.0001	<0.0001
**P value summary**	****	****	****	****
**Significant diff. among means (P < 0.05)?**	Yes	Yes	Yes	Yes
**R squared**	0.6546	0.5645	0.9333	0.736
**Brown-Forsythe test**				
**F (DFn, DFd)**	0.9689 (10, 55)	0.5554 (10, 55)	0.8925 (10, 55)	5.091 (10, 55)
**P value**	0.4805	0.8425	0.5459	<0.0001
**P value summary**	ns	ns	ns	****
**Are SDs significantly different (P < 0.05)?**	No	No	No	Yes
**Bartlett's test**				
**Bartlett's statistic (corrected)**	22.26	8.453	10.4	32.09
**P value**	0.0138	0.5846	0.4063	0.0004
**P value summary**	*	ns	ns	***
**Are SDs significantly different (P < 0.05)?**	Yes	No	No	Yes
